# INCREASING DEMENTIA CARE MASTERY FOR RURAL CAREGIVERS: LESSONS FROM ADAPTING THE HARMONY @ HOME INTERVENTION

**DOI:** 10.1093/geroni/igac059.1548

**Published:** 2022-12-20

**Authors:** Allison Gibson, Laura Henley, Vickie Fairchild, David Goss, Carolyn Baum, Gregory Jicha, Elizabeth Rhodus

**Affiliations:** University of Kentucky, Lexington, Kentucky, United States; HealthPRO Heritage, Louisville, Kentucky, United States; Northeast AHEC, St. Clair Healthcare, Morehead, Kentucky, United States; Northeast AHEC, St. Clair Healthcare, Morehead, Kentucky, United States; Washington University School of Medicine in St. Louis, St. Louis, Missouri, United States; University of Kentucky, Lexington, Kentucky, United States; University of Kentucky, Lexington, Kentucky, United States

## Abstract

Providing care support to rural Alzheimer’s disease and related dementia (ADRD) caregivers has always been challenging, but particularly with the COVID-19 pandemic, new barriers have emerged for families in accessing care support. Delivered via telehealth, Harmony at HOME (H@H) provides dementia care mastery for caregivers of persons with ADRD in the skills of assessing and modifying the home environment to promote "person-environment fit," whereby increasing functional activity engagement and minimizing maladaptive behaviors. To enhance care supports specific to the needs of rural communities, this study reports the findings of two focus groups with rural caregivers who participated in the H@H intervention. Focus groups were aimed to identify the needs of caregivers in rural communities to guide adaptation approaches for future implementation of the H@H intervention in these underserved areas. Additional studies to assess real-world efficacy of the adaptations to H@H are needed as a next step toward implementing this intervention in clinical, home care settings.

